# Association between active commuting and low-grade inflammation: a population-based cross-sectional study

**DOI:** 10.1093/eurpub/ckad213

**Published:** 2023-12-08

**Authors:** Sara Allaouat, Jaana I Halonen, Juuso J Jussila, Pekka Tiittanen, Jenni Ervasti, Tiia Ngandu, Santtu Mikkonen, Tarja Yli-Tuomi, Pekka Jousilahti, Timo Lanki

**Affiliations:** Institute of Public Health and Clinical Nutrition, University of Eastern Finland, Kuopio, Finland; Department of Health Security, Finnish Institute for Health and Welfare, Kuopio and Helsinki, Finland; Department of Health Security, Finnish Institute for Health and Welfare, Kuopio and Helsinki, Finland; Institute of Public Health and Clinical Nutrition, University of Eastern Finland, Kuopio, Finland; Department of Health Security, Finnish Institute for Health and Welfare, Kuopio and Helsinki, Finland; Department of Health Security, Finnish Institute for Health and Welfare, Kuopio and Helsinki, Finland; Finnish Institute of Occupational Health, Helsinki, Finland; Department of Public Health and Welfare, Finnish Institute for Health and Welfare, Helsinki, Finland; Department of Technical Physics, University of Eastern Finland, Kuopio, Finland; Department of Environmental and Biological Sciences, University of Eastern Finland, Kuopio, Finland; Department of Health Security, Finnish Institute for Health and Welfare, Kuopio and Helsinki, Finland; Department of Public Health and Welfare, Finnish Institute for Health and Welfare, Helsinki, Finland; Institute of Public Health and Clinical Nutrition, University of Eastern Finland, Kuopio, Finland; Department of Health Security, Finnish Institute for Health and Welfare, Kuopio and Helsinki, Finland; Department of Environmental and Biological Sciences, University of Eastern Finland, Kuopio, Finland

## Abstract

**Background:**

Prior studies suggest that physical activity lowers circulating C-reactive protein (CRP) levels. However, little is known about the association between regular active commuting, i.e. walking or cycling to work, and CRP concentrations. This study examines whether active commuting is associated with lower CRP.

**Methods:**

We conducted a cross-sectional study using population-based FINRISK data from 1997, 2002, 2007 and 2012. Participants were working adults living in Finland (*n* = 6208; mean age = 44 years; 53.6% women). We used linear and additive models adjusted for potential confounders to analyze whether daily active commuting, defined as the time spent walking or cycling to work, was associated with lower high-sensitivity (hs-) CRP serum concentrations compared with passive commuting.

**Results:**

We observed that daily active commuting for 45 min or more (vs. none) was associated with lower hs-CRP [% mean difference in the main model: −16.8%; 95% confidence interval (CI) −25.6% to −7.0%), and results were robust to adjustment for leisure-time and occupational physical activity, as well as diet. Similarly, active commuting for 15–29 min daily was associated with lower hs-CRP in the main model (−7.4; 95% CI −14.1 to −0.2), but the association attenuated to null after further adjustments. In subgroup analyses, associations were only observed for women.

**Conclusions:**

Active commuting for at least 45 min a day was associated with lower levels of low-grade inflammation. Promoting active modes of transport may lead not only to reduced emissions from motorized traffic but also to population-level health benefits.

## Introduction

Chronic low-grade inflammation is a mild and generally long-lasting inflammatory state characterized by, but not limited to, a slight elevation in levels of circulating inflammatory biomarker C-reactive protein (CRP).^1^ Such elevated CRP levels are associated with major non-communicable diseases, such as cardiovascular disease, causing death and disability worldwide.^2^

CRP has several roles in the immune response, including cytokine and phagocytosis regulation.^3^ It also has both pro-inflammatory and anti-inflammatory properties whenever tissue damage occurs. However, the long-term presence of slightly elevated circulating CRP levels may indicate a failure of the inflammatory response and lead to chronic low-grade inflammation.^4^ High-sensitivity CRP (hs-CRP) tests can help establish the presence or absence of low-grade inflammation, as they have the power to detect CRP plasma values below 10 mg/l.

Evidence is compelling on the potential of physical activity to reduce circulating CRP levels.^5^ Prior studies have suggested that higher exercise intensities and endurance training may lead to the largest benefits, especially in middle-aged or overweight adults.^6^^,^^7^ However, high-intensity interval training seems insufficient to reduce circulating CRP levels, at least in populations with metabolic disorders.^8^

It is also unclear whether active commuting, i.e. walking or cycling to work, alone entails a sufficient level of physical activity to lead to noticeable changes in CRP levels. Few studies have assessed whether regular active commuting is associated with lower CRP levels.^9–14^ At the same time, active commuting may also lead to higher exposure to traffic-related air pollution and noise compared with, e.g. car use.^15^ As air pollution and noise exposure have both been linked to an increase in CRP levels,^16–18^ this may counteract the health benefits of physical activity.

This study examines whether active commuting is associated with lower hs-CRP, independently from other domains of physical activity and despite commute-related air pollution and noise exposure. We hypothesize that active commuters have lower hs-CRP levels compared with passive commuters. The topic is timely because a change from motorized traffic to sustainable means of commuting is urgently needed to mitigate climate change.

## Methods

### Study population

We used the FINRISK study, a population-based survey coordinated by the Finnish Institute for Health and Welfare, to assess chronic and non-communicable disease risk factors in the population living in Finland. The study has been described in detail elsewhere.^19^ The analysis was based on data from the cities of Helsinki and Vantaa (area 1) as well as Turku and Loimaa regions (area 2) for the years 1997 (*n* = 3289), 2002 (*n* = 2626), 2007 (*n* = 2346) and 2012 (*n* = 2264). The eligible participants were aged 25–74 years and received a questionnaire by mail to fill in as well as an invitation to a health examination. The consent of all the participants has been obtained. Ethical permissions were granted from different ethics committees depending on the survey year, including from the ethical committee of the Finnish Institute of Health and Welfare and the Coordinating Ethics Committee for the Helsinki and Uusimaa Hospital District. Corresponding reference numbers were 38/96 (FINRISK 1997), 87/2001 (FINRISK 2002), 229/E0/2006 (FINRISK 2007) and 162/13/03/00/2011 (FINRISK 2012). Eligible participants in this study were employed individuals with complete information on exposure, outcome and covariates included in the main model as described in statistical analysis and results. Key characteristics of the excluded participants are described in the results.

### Exposure variable

Information on active commuting was obtained by asking the participants how many minutes they walk, cycle or exercise to and from work daily. Possible answers were ‘I only use motorized vehicles’, ‘less than 15 min’, ‘15–29 min’, ‘30–44 min’, ‘45–59 min’ and ‘more than 60 min’. We used the first category corresponding to passive commuting as the reference group. Because of the low number of observations, we combined the two last categories into one category representing daily active commuting for 45 min or more. Since active commuting was expressed by the time spent engaging in it, we considered that it is not limited to its physical activity component, but also involves exposure to environmental stressors like noise and air pollution. Indeed, the time that a participant spends in physical activity to commute is the same time in which the participant is exposed to noise and air pollution during commuting.

### Outcome variable

We used serum hs-CRP concentrations as an indicator of low-grade inflammation. The blood samples were drawn during the health examination and the blood analysis methods are described elsewhere.^12^ Because the distribution of the variable residuals was strongly skewed, we log-transformed hs-CRP values for the main analysis. We then used percentual mean difference in hs-CRP serum concentrations for the interpretation of the results.

### Covariates

Based on the literature, plausibility, and data availability, we included age, sex, education level, household income, marital status, smoking status, alcohol consumption and exposure to environmental tobacco smoke as confounders in the main model. We also controlled for occupational physical activity in the main model. On the one hand, we hypothesized that it could be associated with active commuting. For instance, individuals engaged in physically active work might not be willing to include more physical activity during commuting. On the other hand, a possible association between occupational physical activity and CRP has been suggested.^20^ The variables included in the main model were self-reported (for variable descriptions, see Supplementary table S1). To reduce sampling variability and improve precision, we also took the study year/area and neighbourhood low-income rate into account. The latter corresponds to the percentage of inhabitants aged 18 years or older in the lowest-income quintile in their respective postal code area. We also considered other covariates, including medical history of cardiovascular diseases and diabetes, body mass index (BMI), and measurement season, in further analyses. These variables were available for every survey year. Furthermore, leisure-time physical activity and food intake (meat, fish, raw vegetables and fruits) were available for the years 1997, 2002 and 2007, but not for 2012, which is why they were not included in the main models. Exposure to road-traffic NO_2_ was estimated by modelling outdoor concentrations at all residential addresses as described in Supplementary Methods S2.

### Statistical analysis

We analyzed the data cross-sectionally by carrying out three linear regression models using the *glm()* function in *R*.^21^ The basic model included age, sex, study year and area. The intermediate model additionally included education level, household income, smoking status and alcohol consumption. In addition to variables of the intermediate model, the main model included exposure to environmental tobacco smoke, marital status, neighbourhood-level low-income rate and occupational physical activity. We also ran the main model for men and women separately so that we could compare the results with a prior study using the same data.^12^

We then investigated potential multicollinearities between variables using the *vif* function in the *car* package.^22^ We also explored if the confounders used on a continuous scale had a linear relationship with hs-CRP using the *gam* function and smoothing terms in the *mgcv* package.^23^ We judged that age was not linear, but household income and BMI were. Therefore, we ran the main analyses and additional analyses with a smoothing term for age. In addition, we tested residuals for normality, and the effect of influential datapoints based on the hat matrix, which allows to convert observed values to fitted values using the least squares method.^24^ Related procedures are described in detail in Supplementary material (R code S3). All these diagnostic tests were run on the main model.

We carried out additional analyses where we separately added BMI, then history of cardiovascular disease and diabetes to the main model. We did not include a medical history of cardiovascular diseases and diabetes, or BMI, in the main analyses because they can both confound and mediate the studied association. We also conducted different sensitivity analyses on the main model. First, we adjusted the main model for additional lifestyle factors (i.e. leisure-time physical activity, and fruit, vegetable, meat, and fish intake). Second, we adjusted the model for residential exposure to road-traffic NO_2_. Third, we adjusted for measurement season in the main model. Fourth, we only included participants whose serum hs-CRP levels were below 10 mg/l as higher values would indicate acute inflammation or infection. Finally, we excluded year 1997, which was the period with the highest levels of air pollution, to make the results more representative of the present-day air pollution levels.^25^

For all the analyses, we used *R* software version 4.2.1 and *RStudio* version 1.4.1106.0. Results are presented as percentage differences in mean hs-CRP serum levels between active commuting categories and their 95% confidence intervals.

## Results

For the survey years and areas used in this study, the participation rate varied between 56% and 68% in men, and between 61% and 75% in women, totalling 10525 participants. For this study, we only included employed individuals (*n* = 6763). From these, we further excluded participants with missing data on the exposure, associated covariates in the main model, or the outcome. The final analytical sample included 6208 participants (table 1).

**Table 1:** Characteristics of the Finnish residents who participated in the FINRISK study surveys of 1997, 2002, 2007 and 2012 (*n* = 6208)

Categorical variables			
Characteristic	Variable	Category	*n* (%)
Study information (*n* = 6208)	Study year	1997	1750 (28.2)
		2002	1729 (27.9)
		2007	1414 (22.8)
		2012	1315 (21.2)
	Area	Area 1	3106 (50.0)
		Area 2	3102 (50.0)
	Measurement season	Winter	3461 (55.8)
		Spring	2747 (44.2)
Socio-demographic information (*n* = 6208)	Sex	Female	3330 (53.6)
		Male	2878 (46.4)
	Education level	Primary education	1001 (16.1)
		Secondary education	3716 (59.9)
		Higher education	1491 (24.0)
	Marital status	Single	1107 (17.8)
		Married or living with a partner	4369 (70.4)
		Divorced, separated, or widowed	732 (11.8)
	Household income	Low	1607 (25.9)
		Medium	2634 (42.4)
		High	1967 (31.7)
Lifestyle information	Smoking status	Current smoker	1726 (27.8)
	(*n* = 6208)	Ex-smoker	1638 (26.4)
		Non-smoker	2844 (45.8)
	Exposure to environmental tobacco smoke	No	5392 (86.9)
	(*n* = 6208)	Yes	816 (13.1)
	Alcohol consumption	Never	1484 (23.9)
	(*n* = 6208)	1–3 drinks a week	1386 (22.3)
		3–6 drinks a week	1107 (17.8)
		More than 6 drinks a week	2231 (35.9)
	Raw vegetable intake	Daily	3414 (55.0)
	(*n* = 4886)	Weekly	1218 (19.6)
		Seldom or never	254 (4.1)
	Fruit intake	Daily	2976 (47.9)
	(*n* = 4884)	Weekly	1484 (23.9)
		Seldom or never	424 (6.8)
	Meat intake	Daily	4169 (67.2)
	(*n* = 4888)	Weekly	508 (8.2)
		Seldom or never	211 (3.4)
	Fish intake	Daily	134 (2.2)
	(*n* = 4837)	Weekly	3092 (49.8)
		Seldom or never	1611 (26.0)
	Daily active commuting	Not at all	2160 (34.8)
	(*n* = 6208)	<15 min	1292 (20.8)
		15–29 min	1548 (24.9)
		30–44 min	728 (11.7)
		45 min or more	480 (7.7)
	Occupational physical activity	Inactive	2780 (44.8)
	(*n* = 6208)	Lightly active	1944 (31.3)
		Moderately active	1222 (19.7)
		Very active	262 (4.2)
	Leisure-time physical activity	Inactive	1293 (20.8)
	(*n* = 4893)	Lightly active	1061 (17.1)
		Moderately active	2027 (32.7)
		Very active	512 (8.2)
Health-related information	Diabetes	No	5980 (96.3)
	(*n* = 6155)	Yes	175 (2.8)
	Cardiovascular disease	No	4042 (65.1)
	(*n* = 6129)	Yes	2087 (33.6)
Continuous variables			
Variable (unit, *n*)		Mean (SD)	Median (min, max)
Age (year, 6208)		44 (11)	44 (25, 74)
BMI (kg/m², 4893)		26 (4)	25 (15, 49)
Neighbourhood low-income rate (%, 6208)		17.9 (4.5)	17.0 (6.8, 48.7)
Road-traffic NO_2_ (µg/m^3^)		8.30 (4.18)	7.73 (0.10, 27.74)
hs-CRP (mg/l, 6208)		2.02 (4.32)	0.88 (0.10, 94.05)

Notes: BMI, body mass index; hs-CRP, high-sensitivity C-reactive protein; NA, not available; SD, standard deviation.

The employed individuals who were excluded (*n* = 555) were slightly older (mean age 46 vs. 44 years) and had a slightly higher BMI (mean BMI 27 vs. 26 kg/m^2^) and hs-CRP (median 0.98 vs. 0.88 mg/l). They were to some extent less educated (21% vs. 24% in higher education) and less often married or living with a partner (65% vs. 70%). A greater share of them was smoking (32% vs. 28%), but they were to some extent consuming less alcohol (31% vs. 36% drinking more than six drinks weekly).

Compared with participants who were inactive in their leisure time (7.0%), ‘very active’ individuals (10.9%) belonged to the highest active commuting category (≥45 min) more often. Similarly, people engaging in active commuting for at least 45 min daily (11.7%) were more often ‘very active’ in their leisure time compared with passive commuters (8.5%). Moreover, 9.5% (*n* = 317) of women engaged in active commuting for 45 min or more daily against 5.7% of men (*n* = 163). Women were also to some extent more physically active in their leisure time, with 8.8% of women (*n* = 293) and 7.6% of men (*n* = 219) being ‘very active’. In addition, the mean BMI in women was 26 kg/m^2^ (*n* = 3330) against 27 kg/m^2^ in men (*n* = 2878), and women had less cardiovascular disease history (26.4%, *n* = 879) than men (42.0%, *n* = 1208).

Table 2 represents the main results for the associations between active commuting and hs-CRP. In all the models, except the model including men only, daily active commuting lasting at least 45 min was associated with lower serum hs-CRP concentrations compared with passive commuters. Removing the most influential datapoints with hat value >0.01 (*n* = 47) did not affect the results.

**Table 2:** Association between active commuting and high-sensitivity C-reactive protein (hs-CRP) among Finnish residents who participated in the FINRISK study surveys of 1997, 2002, 2007 and 2012

		Difference (%) in hs-CRP mean serum concentration (vs. reference group) with 95% confidence intervals			
Time spent daily in active commuting	Passive commuting (reference)	<15 min	15–29 min	30–44 min	≥45 min
Basic model^a^	–	0.9 (−6.6 to 9.0)	−8.7 (−15.3 to −1.6)*	−7.0 (−15.4 to 2.3)	−17.4 (−26.1 to −7.6)*
(*n* = 6208)					
Intermediate model^b^	–	1.6 (−6.0 to 9.7)	−7.2 (−13.9 to 0.0)	−5.2 (−13.8 to 4.3)	−16.6 (−25.3 to −6.8)*
(*n = *6208)					
Main model^c^	–	1.5 (−6.1 to 9.6)	−7.4 (−14.1 to −0.2)*	−5.4 (−14.0 to 4.0)	−16.8 (−25.5 to −7.0)*
(*n* = 6208)					
Women^d^	–	−0.3 (−11.3 to 12.1)	−9.5 (−18.5 to 0.5)	−5.2 (−16.5 to 7.7)	−23.1 (−33.6 to −10.9)*
(*n = *3330)					
Men^e^	–	2.9 (−7.0 to 13.9)	−4.8 (−14.8 to 6.3)	−7.8 (−20.7 to 7.1)	−4.4 (−19.7 to 13.8)
(*n = *2878)					

a Basic model adjusted for age, sex, study year and area.

b Intermediate model additionally adjusted for education level, smoking status, alcohol consumption and household income.

c Main model additionally adjusted for marital status, exposure to environmental tobacco smoke, occupational physical activity and neighbourhood low-income rate.

d Main model including women only.

e Main model including men only.

* *P*<0.05.

Table 3 represents the results of the additional analyses in which the association for 45 min or more remained robust.

**Table 3:** Association between active commuting and high-sensitivity C-reactive protein (hs-CRP) among Finnish residents who participated in the FINRISK study surveys of 1997, 2002, 2007 and 2012: additional analyses

		Difference (%) in hs-CRP mean serum concentration (vs. reference group) with 95% confidence intervals			
Time spent daily in active commuting	Passive (reference)	<15 min	15–29 min	30–44 min	45 min or more
BMI added^a^ (*n = *6208)	–	2.9 (−4.2 to 10.5)	−4.4 (−10.8 to 2.5)	−1.7 (−10.0 to 7.3)	−11.6 (−20.2 to −2.1)*
Cardiovascular diseases and diabetes added^b^ (*n = *6106)	–	2.1 (−5.5 to 10.3)	−7.0 (−13.8 to 0.3)	−4.4 (−13.1 to 5.2)	−16.1 (−25.0 to −6.2)*
Additional lifestyle variables^c^ (*n = *4828)	–	0.7 (−7.6 to 9.7)	−1.9 (−9.7 to 6.6)	−3.5 (−13.2 to 7.2)	−15.4 (−25.2 to −4.3)*
Air pollution variable added^d^ (*n = *5216)	–	4.5 (−4.0 to 13.7)	−6.3 (−13.7 to 1.8)	−3.1 (−12.6 to 7.4)	−16.3 (−25.8 to −5.7)*
Measurement season included^e^ (*n = *6208)	–	2.3 (−5.3 to 10.5)	−5.1 (−11.9 to 2.2)	−3.2 (−12.0 to 6.4)	−14.8 (−23.7 to −4.9)*
hs-CRP >10 mg/l excluded^f^ (*n = *6050)	–	2.6 (−4.5 to 10.3)	−4.6 (−11.0 to 2.2)	−1.8 (−10.0 to 7.3)	−13.3 (−21.8 to −4.0)*
Year 1997 excluded^g^ (*n = *4458)	–	4.0 (−5.1 to 14.0)	−6.7 (−14.7 to 2.0)	−0.5 (−11.3 to 11.6)	−14.0 (−24.6 to −2.0)*

Notes: BMI, body mass index; hs-CRP; high-sensitivity C-reactive protein; NO_2_, nitrogen dioxide.

a Main model additionally adjusted for BMI.

b Main model additionally adjusted for history of cardiovascular disease and diabetes.

c Main model additionally adjusted for leisure-time physical activity, meat, fish, fruit and vegetable consumption.

d Main model additionally adjusted for residential exposure to road-traffic NO_2_.

e Main model additionally adjusted for measurement season.

f Main model with participants having hs-CRP >10 mg/l removed.

g Main model with FINRISK 1997 study participants removed.

* *P* < 0.05.

## Discussion

We observed that Finnish adults engaging in high levels of daily active commuting to work (at least 45 min a day) had lower serum hs-CRP concentrations compared with passive commuters. Adjusting for key confounders, including occupational and leisure-time physical activity, diet, and residential exposure to road-traffic NO_2_, as well as additional factors such as BMI or history of cardiovascular disease and diabetes, did not substantially affect the results. No robust associations were observed for lower levels of active commuting. Further, in the subgroup analysis, the association between active commuting and hs-CRP was only observed for women.

Active commuting is one way to be physically active, which can decrease hs-CRP. We controlled for both leisure-time and occupational physical activity in the analyses, which turned out to be important because people exercising in their free time were also more likely to use active transportation modes in this study. Similar findings have been previously reported.^26^ Furthermore, to our knowledge, only one prior study controlled for leisure-time physical activity.^11^ The cross-sectional study reported that individuals engaging in active commuting for <150 min weekly had significantly lower odds of high hs-CRP levels compared with passive commuters.^11^ While associations in our study were non-significant at this low level of active commuting, the effect estimates were in the same direction. Unlike in our study, higher levels of active commuting were not associated with hs-CRP concentrations in the above-mentioned study. The differences could be explained by the relatively smaller sample size compared with our study.

Our findings align with those from some prior studies that considered the time spent engaging in active commuting. In a small Danish trial, physically inactive, healthy, overweight or obese participants were assigned to a 6-month bicycle commuting intervention. Women had to bike 9–15 km and men 11–17 km daily. These distances were calculated so that related energy expenditures would be in line with the physical activity guidelines (150 min of weekly moderate-intensity physical activity). The trial showed a 30% decrease in the cyclists’ CRP levels during the 6-month cycling period.^9^^,^^27^ Furthermore, a Brazilian cross-sectional study found that low-grade inflammation tended to be lower in individuals engaging in active commuting for more than 150 min weekly compared with individuals who spend less time in active commuting.^10^ Some studies reported no association between the time spent in active commuting and CRP.^13^^,^^14^ Similarly, no associations were found in studies considering commuting physical activity as defined by metabolic equivalent of task.^20^^,^^28^^,^^29^ However, many of these studies found that total physical activity that comprised active commuting as one of the physical activity domains was associated with lower circulating CRP concentrations,^13^^,^^20^^,^^28^ while other domains such as leisure-time physical activity were not necessarily associated with CRP when considered separately.^20^^,^^28^

In subgroup analyses, the association between daily active commuting and serum hs-CRP concentration was observed among women but not men. These results are in line with the earlier study using FINRISK data.^12^ One explanation could be that women in our study were in the highest active commuting category more often than men. They also tended to be more physically active in their leisure time, had lower BMI and fewer cardiovascular diseases.

This study examined the net effect of active commuting on hs-CRP, i.e. the combined effect of beneficial changes from physical activity and harmful changes due to environmental exposures. Indeed, commuters and especially active commuters may be exposed to high levels of traffic-related air pollution and noise during their journey.^15^ Even though active commuters can be less exposed in some study areas compared with passive commuters, the evidence shows that inhaled dose of air pollution might still be higher in active commuters.^30^ In both cases, this would favour a CRP increase because even short-term exposures to air pollution during commuting could lead to increased low-grade inflammation.^31^ Our results were robust after controlling for residential exposure to road-traffic NO_2_. We did the adjustment because residential NO_2_ exposure has been observed to correlate with NO_2_ exposure during commuting.^32^^,^^33^ Thus, active commuting for 45 min or more might have a beneficial, anti-inflammatory effect on Finnish adults, despite such exposures. Furthermore, active commuting for <45 min a day seems not to be detrimental regarding inflammation. These results are in line with a study where air pollution exposure did not seem to affect circulating CRP levels in cyclists.^34^ Moreover, health impact assessments also suggest that the net effect of active commuting is beneficial even with detrimental exposures.^35^

Different biological mechanisms could explain the potential effect of regular physical activity in reducing low-grade systemic inflammation. For example, a direct reduction in fat mass could disfavour the secretion of pro-inflammatory cytokines, such as interleukin (IL)-6, tumour necrosis factor (TNF) and leptin from adipose tissue.^5^^,^^36^ It could also favour the secretion of anti-inflammatory cytokines, including adiponectin. In addition, IL-10 is known to increase after acute physical activity. This would increase insulin sensitivity and reduce sustained inflammatory state in adipose tissue by reducing macrophage infiltration. Regular exercise also shifts the balance of macrophage recruitment from pro-inflammatory to anti-inflammatory phenotypes, which is probably due to a lowered level of tissue inflammation. Furthermore, depending on its intensity and duration, exercise induces the release of cortisol known for its anti-inflammatory effects. It is also involved in the release of catecholamines that inhibit the secretion of pro-inflammatory TNF and IL-1β, for instance. Lastly, regular exercise improves endothelial function by reducing local inflammation.^5^^,^^36^

Besides the cross-sectional design, the study has some other limitations. For example, we did not control for residential noise exposure even when it might be correlated with commute-related noise exposure (and associated with higher CRP).^17^^,^^18^^,^^37^ We were not able to adjust for greenspace exposure. Greenspace has been associated with lower circulating hs-CRP levels^38^ and may also be associated with commuting behaviour. In one study, living near green areas was associated with lower levels of active commuting, perhaps because of urban sprawl and long commuting journeys.^39^ This makes it unclear whether it would have affected our results. In addition, we could not differentiate between walking and cycling to work; thus, it is unclear whether low-intensity walking alone would decrease CRP levels. Besides, serum hs-CRP was measured only once, while this biomarker has a significant intra-individual variability.^40^ Any subsequent misclassification is likely to bring the association between active commuting and hs-CRP towards the null. Consequently, we could have missed an association between active commuting for <45 min daily and hs-CRP.

This study suggests that high level of daily active commuting, 45 min or more, is associated with lower levels of low-grade inflammation, which may reduce associated disease risks and complications. The association seems to be independent of leisure-time and occupational physical activity. Because of the population-based design, the robustness of the findings and their alignment with some prior research findings, the results can be assumed to be generalizable to other working-age populations, especially in areas where air pollution and noise exposures during commuting are relatively low. Therefore, in addition to climate change mitigation, active commuting could lead to public health benefits.

## Supplementary Material

ckad213_Supplementary_Data

## Data Availability

The ‘National FINRISK Study 1992-2012’ data is available upon application to THL biobank at https://thl.fi/en/web/thl-biobank/for-researchers/sample-collections/the-national-finrisk-study-1992-2012 (accessed 1 November 2023). More information: finriski(at)thl.fi. Key points Regular leisure-time physical activity lowers low-grade inflammation, including C-reactive protein (CRP). Little is known about whether active commuting, i.e walking or cycling to work has a similar effect. Compared with passive commuting, daily active commuting for 45 min or more was associated with lower CRP. The association was robust even after adjusting for different physical activity domains and diet. Promoting active commuting could help reduce many chronic diseases, while mitigating climate change.
