# Supplementary material for: Association between active commuting and low-grade inflammation: a population-based cross-sectional study
Source: Eur J Public Health. 2023 Dec 8;34(2):292–8. doi: 10.1093/eurpub/ckad213 (PMC10990550; doi:10.1093/eurpub/ckad213)
Supplement: ckad213_Supplementary_Data [file ckad213_supplementary_data.pdf]

Association between active commuting and low-grade inflammation: a population-based cross-sectional study

Supplemental material

Sara Allaouat[1,2], corresponding author, University of Eastern Finland, Institute of Public Health and Clinical Nutrition, P.O. Box 1627, FI-70211 Kuopio, Finland, sara.allaouat@uef.fi

Jaana I. Halonen[2]

Juuso J. Jussila[1,2]

Pekka Tiittanen[2]

Jenni Ervasti[3]

Tiia Ngandu[4]

Santtu Mikkonen[5,6]

Tarja Yli-Tuomi[2]

Pekka Jousilahti[4]

Timo Lanki[1,2,6]

[1] Institute of Public Health and Clinical Nutrition, University of Eastern Finland, Kuopio, Finland

[2] Department of Health Security, Finnish Institute for Health and Welfare, Kuopio and Helsinki, Finland

[3] Finnish Institute of Occupational Health, Helsinki, Finland

[4] Department of Public Health and Welfare, Finnish Institute for Health and Welfare, Helsinki, Finland

[5] Department of Technical Physics, University of Eastern Finland, Kuopio, Finland

[6] Department of Environmental and Biological Sciences, University of Eastern Finland, Kuopio, Finland

Table S1

Variable description

**Categorical variable (variable name in the dataset)**	**Self-reported survey questions**	**Categories used in the statistical models**
FINRISK study year (study)	NA	1 = 1997<br>2 = 2002<br>3 = 2007<br>4 = 2012
Study area (area)		0 = area 1<br>1 = area 2
Measurement season (season)<br>NB: built from visit_month (winter = January and February, spring = March to May) according to participation period		1 = winter<br>2 = spring
Sex (sex_b)	*Gender*	0 = female<br>1 = male
Education level (edulev_b)	*What is your education?*	1 = primary education<br>2 = secondary education<br>3 = higher education
Marital status (mar_stat_b)	*What is your marital status?*	1 = single

		2 = married/with partner<br>3 = divorced/separated or widowed
Household income (income_b)	*How large was your household's income last year (before tax deduction)?*	1 = low<br>2 = medium<br>3 = high
Smoking status (smoking_b)	*Have you ever smoked?*<br>*Do you smoke now?*<br><br>In the 2002,2007, and 2012 surveys, one question was more detailed:<br>*Do you smoke now (cigarettes, cigars, pipefuls)?*	1 = current smokers<br>2 = ex-smokers<br>3 = never smokers
Exposure to environmental tobacco smoke (etsc_b_fin)	*How many hours do you daily spend in spaces where you have to inhale other people's smoke?*<br><br>In the 2002, 2007, and 2012 surveys, the specification of spaces was requested:<br>• *at work .. hours*<br>• *at home .. hours*<br>• *other places .. hours*<br><br>In the 2007 and 2012 surveys, the question was more detailed:<br>*How many hours do you daily spend in indoor spaces where you have to inhale other people's smoke?*	0 = no<br>1 = yes

Alcohol consumption<br>(totalalc_week_cat_b_fin)	*Have you during the past year (last 12 months) had any alcohol (beer, wine or spirits)?*<br>*How many glasses (restaurant measures) or bottles did you drink during the last week (last 7 days) of the following (If not at all, mark 0)*<br>Depending on the survey year, a drink referred to one of the following options.<br>• 1/3-liter bottle/can of beer (class III) or medium strong cider (sold in markets, alcohol content 2.9-4.7%)<br>• 1/3-liter bottle/can of beer (class IV, alcohol content over 4.7%)<br>• 1/3-liter bottle/can of strong cider or long drinks (sold only in the ALKO stores, alcohol content over 4.7%)<br>• restaurant measures (c. 4 cl) of spirits or other strong alcohol<br>• 1 glass (c. 12 cl) of cider (alcohol c. 5 %), wine, or the equivalent (alcohol over 5 %)	1 = never<br>2 = 1-3 drinks/week<br>3 = 3-6 drinks/week<br>4 = > 6 drinks/week
Raw vegetable intake<br>(rawveg_b)	*How often do you usually eat the following foods? Think of the past year (last 12 months).*	1 = daily<br>2 = weekly<br>3 = seldom or never
Fruit intake (fruit_b)		1 = daily<br>2 = weekly<br>3 = seldom or never

Meat intake (meat_b)		1 = daily<br>2 = weekly<br>3 = seldom or never
Fish intake (fish_b)		1 = daily<br>2 = weekly<br>3 = seldom or never
Daily active commuting (Q58)	*How many minutes do you walk, ride on a bicycle or otherwise exercise to get to work? (Please count in both traveling to and from work.)*	1 = not at all<br>2 = less than 15 minutes<br>3 = 15-29 minutes<br>4 = 30-44 minutes<br>5 = 45 minutes or more
Occupational physical activity (phys_work_b)	*How demanding is your work physically?*	1 = inactive<br>2 = lightly active<br>3 = moderately active<br>4 = very active
Leisure-time physical activity (physact_w5a2_b)	<u>1997 and 2002 surveys</u><br>*How often do you exercise in your leisure time for at least half an hour so that you at least are mildly out of breath and sweaty?*<br>*How many times in a week do you exercise in your free time for at least half an hour so that you at least are mildly out of breath and sweaty?*	1 = inactive<br>2 = lightly active<br>3 = moderately active<br>4 = very active

	How long does your usual leisure time activity take?<br><br>2007 survey<br>*How often do you in your leisure time exercise for at least 20 minutes so that you at least are mildly out of breath and sweaty (the exercise of travelling to and from work not included)?*<br>*How long does your usual leisure time activity take?*	
Diabetes (diab_b)	Personal history of disease diagnosis (or treatment) as defined by the variable	0 = no<br>1 = yes
Cardiovascular disease (cvd_cat)<br>NB: built from key variables on cardiovascular disease i.e., heart failure (hf_b), ischemic heart disease (ihd_b), cardiovascular disease (cvd_w5a1_b) and coronary heart disease (chd_b_fin)		0 = no<br>1 = yes

Supplemental Methods S2

Estimates of road-traffic NO2 concentrations in the study areas were based on emission and dispersion modelling which used vehicular exhaust emission factors from 2014 (Helsinki and Vantaa) and 2007 (Turku) and the road network (CAR-FMI) dispersion model (1,2). The distances between the receptor points varied from 25 m near the roads to 500 m in rural areas. In the present study, the NO2 concentrations were assigned to participants' homes as the estimated NO2 concentrations at the nearest outdoor modelling point to each home. In the dispersion model, meteorological data of years 2012-2014 for Helsinki and Vantaa, and years 2005-2007 for Turku were used. The highest annual average values for the modelled years were used as the exposure estimates.

References

1. Hannuniemi H, Salmi J, Rasila T, Wemberg A, Komppula B, Lovén K, et al. Pääkaupunkiseudun päästöjen leviämismalliselvitys. Autoliikenteen, energiantuotannon, laivaliikenteen ja lentoliikenteen typenoksidi-, pienhiukkas- ja rikkidioksidipäästöjen leviämismallinnus (Study of the dispersion of emissions in the Helsinki Metropolitan Area. Dispersion modelling of nitrogen oxides, fine particulate matter and sulphur dioxide emissions from road transport, energy production, shipping, and aviation) [Internet]. Helsinki, Finland: Finnish Meteorological Institute, Expert Services, Air Quality and Energy; 2016 May [cited 2023 Nov 14] p. 76. Available from: https://www.hsy.fi/globalassets/ilmanlaatu-ja-ilmasto/tiedostot/pks_ilmanlaaturaportti_2016_2.pdf

2. Salmi J, Lappi S, Rasila T, Lovén K, Hannuniemi H. Turun seudun päästöjen leviämismalliselvitys energiantuotannon, teollisuuden, laivaliikenteen ja autoliikenteen typenoksidi-, rikkidioksidi- ja hiukkaspäästöjen leviämislaskelmat (Turku region emission dispersion modelling study. Dispersion modelling calculations of nitrogen oxide, sulphur dioxide and particle emissions from energy production, industry, ship traffic and road traffic) [Internet]. Helsinki, Finland: Finnish Meteorological Institute, Air Quality Expert Services; 2009 Dec [cited 2023 Nov 14] p. 105. Available from: http://expo.fmi.fi/aqes/public/Turun_seudun_leviamismallilaskelmat_2010.pdf

R Code S3

The code below was used in this study for the main analysis.

```
# MAIN ANALYSIS
```{r}

library(mgcv)

# Basic model adjusted for age, sex, study year and area

basic <-gam(log_crp ~ Q58 + s(age_b) + sex_b + study + area, na.action = na.omit, data = std4)

#summary(basic)

perc.diff <- (exp(coefficients(basic)[2:5])-1)*100 # percentual (%) difference in CRP between each category of active commuting to work and the reference category of no active commuting to work

MD <- coef(basic)[2:5]  # difference in log CRP between each category of active commuting to work and the reference category of no active commuting to work

SE <- summary(basic)["se"]        # extraction of standard error (SE) values

SE <- as.data.frame(SE)

SE[2:5,]

marginoferror <- 1.96*SE[2:5,]       # calculation of margin of error

lwr <- (exp(MD - marginoferror)-1)*100           # calculation of the lower bound of the 95% confidence interval expressed as percentual difference in CRP

upr <- (exp(MD + marginoferror)-1)*100           # calculation of the upper bound of the 95% confidence interval expressed as percentual difference in CRP

basicmodel <- (round(cbind(perc.diff, lwr,upr), 1))         # model results
```
```

Intermediate model additionally adjusted for education level, smoking status, alcohol consumption and household income

For a detailed explanation of the code, please see basic model above

```
intermediate <-gam(log_crp ~ Q58 + s(age_b) + sex_b + study + area+ edulev_b + smoking_b+
totalalc_week_cat_b_fin + income_b, na.action = na.omit, data = std4)

#summary(intermediate)

perc.diff <- (exp(coefficients(intermediate)[2:5])-1)*100

MD <- coef(intermediate)[2:5]

SE <- summary(intermediate)["se"]

SE <- as.data.frame(SE)

SE[2:5,]

marginoferror <- 1.96*SE[2:5,]

lwr <- (exp(MD - marginoferror)-1)*100

upr <- (exp(MD + marginoferror)-1)*100

intermediatemodel <- (round(cbind(perc.diff, lwr,upr), 1))
```

Main model additionally adjusted for exposure to environmental tobacco smoke, marital status, occupational physical activity, and neighborhood income compared to the intermediate model

For a detailed explanation of the code, please see basic model above

```
main <-gam(log_crp ~ Q58 + s(age_b) + sex_b + study + area+ edulev_b +smoking_b+  totalalc_week_cat_b_fin
+ income_b +etsc_b_fin+ mar_stat_b + phys_work_b+ incomearealow, na.action = na.omit, data = std4)

#summary(main)

perc.diff <- (exp(coefficients(main)[2:5])-1)*100
```

MD <- coef(main)[2:5]

SE <- summary(main)["se"]

SE <- as.data.frame(SE)

SE[2:5,]

marginoferror <- 1.96*SE[2:5,]

lwr <- (exp(MD - marginoferror)-1)*100

upr <- (exp(MD + marginoferror)-1)*100

mainmodel <- (round(cbind(perc.diff, lwr,upr), 1))

Summary table for all three models

Table3 <- as.data.frame(cbind(basicmodel, intermediatemodel,mainmodel))

Table3

NB: The smoothing term s() is used on age in all three models because age was found to have a non-linear relationship with CRP in the model diagnostics below

```

# MODEL DIAGNOSTICS

```{r}

# 1. Multicollinearities using the vif function

#install.packages("car")

library(car)

```
mainvif <- glm(log_crp ~ Q58 + age_b + sex_b + study + area+ edulev_b +smoking_b+ totalalc_week_cat_b_fin + income_b +etsc_b_fin+ mar_stat_b + phys_work_b+ incomearealow, na.action = na.omit, family =gaussian, data = std4)

vif(mainvif)

#If the value of GVIF^(1/(2*Df)) is above 2 then there may be collinearity between explanatory variables in the model.

2. Linearity of the confounders used on a continuous scale using the smoothing term s() on these confounders

#library(mgcv)

linearity <- gam(formula = log_crp ~ Q58 + s(age_b) + sex_b + study + area+ edulev_b +smoking_b+ totalalc_week_cat_b_fin + income_b +etsc_b_fin+ mar_stat_b + phys_work_b+ s(incomearealow)+s(bmi_b), na.action = na.omit, family =gaussian, data = std4)

#summary(linearity)

plot(linearity)

Age has a non-linear relationship with CRP

3. Testing residuals for normality

#plot(main)

qq.gam(linearity)
```

```
gam.check(linearity)

# residuals are normally distributed


# 4. Testing the effect of influential datapoints

maininflu <- gam(log_crp ~ Q58 + s(age_b) + sex_b + study + area+ edulev_b +smoking_b+
totalalc_week_cat_b_fin + income_b +etsc_b_fin+ mar_stat_b + phys_work_b+ incomearealow, na.action =
na.omit, family =gaussian, data = std4)


plot(influence(maininflu)) # checks the most influential datapoints according to their hat values

which(influence(maininflu) > 0.01) # gives datapoints having a hat value > 0.01

 # rows 300 1739 1741 1742 1743 1746 2034 2692 2747 2869 2898 2913 3036 3052 3080 3117 3139 3142
3156 3229 3248 3251 3617 3811 3971 4209 4234 4246 4501 4641 4745 4789 4796 4807 4861 4873 4885 4939
5185 5223 5352 5488 5533 5548 5561 5567 5569 5579 5666 5669 5721 5766 5795 5889 5973 6033 6064 6203
---> hat value > 0.01

#install.packages("broom")

library(broom)

library(mgcv)

# augmenting the data (creating a counterfactual dataset that considers the effect of the most influential
datapoints in the main model)

augmented <- as.data.frame(augment_columns(maininflu, data = std4))


nrow(augmented) #the dataset augmented has 6208 rows
```

```
# removing the most influential datapoints based on their hat value

augmented <- augmented[-c(300, 1739, 1741, 1742, 1743, 1746, 2034, 2692, 2747, 2869, 2898, 2913, 3036,
3052, 3080, 3117, 3139, 3142, 3156, 3229, 3248, 3251, 3617, 3811, 3971, 4209, 4234, 4246, 4501, 4641, 4745,
4789, 4796, 4807, 4861, 4873, 4885, 4939, 5185, 5223, 5352, 5488, 5533, 5548, 5561, 5567, 5569, 5579, 5666,
5669, 5721, 5766, 5795, 5889, 5973, 6033, 6064, 6203),]

nrow(augmented) # the dataset augmented now has 6150 rows


## For a detailed explanation of the code used in the model below, please see basic model in the main analysis
above

maininflu_aug <- gam(log_crp ~ Q58 + s(age_b) + sex_b + study + area+ edulev_b +smoking_b+
totalalc_week_cat_b_fin + income_b +etsc_b_fin+ mar_stat_b + phys_work_b+ incomearealow, na.action =
na.omit, family =gaussian, data = augmented)

#summary(maininflu_aug)

perc.diff <- (exp(coefficients(maininflu_aug))-1)*100

MD <- coef(maininflu_aug)

SE <- summary(maininflu_aug)["se"]

SE <- as.data.frame(SE)

SE

marginoferror <- 1.96*SE

lwr <- (exp(MD - marginoferror)-1)*100

upr <- (exp(MD + marginoferror)-1)*100

Maininflu_aug <- (round(cbind(perc.diff, lwr,upr), 1))
```

~~~
```

# SENSITIVITY ANALYSIS
```{r}

## For a detailed explanation of the code used in every model, please see basic model in the main analysis above

#library(mgcv)

#1. Include cvd and diabetes (diab_b)

disease <-gam(log_crp ~ Q58 + s(age_b) + sex_b + study + area+ edulev_b +smoking_b+
totalalc_week_cat_b_fin + income_b +etsc_b_fin+ mar_stat_b + phys_work_b+ incomearealow+cvd_+diab_b,
na.action = na.omit, data = std4)

#summary(disease)

perc.diff <- (exp(coefficients(disease)[2:5])-1)*100

MD <- coef(disease)[2:5]

SE <- summary(disease)["se"]

SE <- as.data.frame(SE)

SE[2:5,]

marginoferror <- 1.96*SE[2:5,]

lwr <- (exp(MD - marginoferror)-1)*100

upr <- (exp(MD + marginoferror)-1)*100
~~~

```
Disease <- (round(cbind(perc.diff, lwr,upr), 1))

#2. Include BMI (bmi_b)

bmi <-gam(log_crp ~ Q58 +s(age_b) + sex_b +study + area+ edulev_b +smoking_b+  totalalc_week_cat_b_fin +
income_b +etsc_b_fin+ mar_stat_b + phys_work_b+ incomearealow + bmi_b, na.action = na.omit, data = std4)

#summary(bmi)
perc.diff <- (exp(coefficients(bmi)[2:5])-1)*100
MD <- coef(bmi)[2:5]
SE <- summary(bmi)["se"]
SE <- as.data.frame(SE)
SE[2:5,]
marginoferror <- 1.96*SE[2:5,]
lwr <- (exp(MD - marginoferror)-1)*100
upr <- (exp(MD + marginoferror)-1)*100
BMI <- (round(cbind(perc.diff, lwr,upr), 1))

#3. Exclude high CRP values (>=10mg/L)

exchighcrp1 <- filter(std4, crp_b<10)
```

```
lowcrp <-gam(log_crp ~ Q58 + s(age_b) + study + area+ edulev_b +smoking_b+  totalalc_week_cat_b_fin +
income_b +etsc_b_fin+ mar_stat_b + phys_work_b+ incomearealow, na.action = na.omit, data = exchighcrp1)

#summary(lowcrp)

perc.diff <- (exp(coefficients(lowcrp)[2:5])-1)*100

MD <- coef(lowcrp)[2:5]

SE <- summary(lowcrp)["se"]

SE <- as.data.frame(SE)

SE[2:5,]

marginoferror <- 1.96*SE[2:5,]

lwr <- (exp(MD - marginoferror)-1)*100

upr <- (exp(MD + marginoferror)-1)*100

Lowcrp <- (round(cbind(perc.diff, lwr,upr), 1))


#4. Include participation month (season)

season <-gam(log_crp ~ Q58 + s(age_b) + study + area+ edulev_b +smoking_b+  totalalc_week_cat_b_fin +
income_b +etsc_b_fin+ mar_stat_b + phys_work_b+season + incomearealow, na.action = na.omit, data = std4)

#summary(season)

perc.diff <- (exp(coefficients(season)[2:5])-1)*100

MD <- coef(season)[2:5]

SE <- summary(season)["se"]

SE <- as.data.frame(SE)

SE[2:5,]
```

```
marginoferror <- 1.96*SE[2:5,]

lwr <- (exp(MD - marginoferror)-1)*100

upr <- (exp(MD + marginoferror)-1)*100

Season <- (round(cbind(perc.diff, lwr,upr), 1))



#5. Exclude year 1997

exc97 <- filter(std4, study!=1997)

no97 <-gam(log_crp ~ Q58 + s(age_b) + study + area+ edulev_b +smoking_b+  totalalc_week_cat_b_fin +
income_b +etsc_b_fin+ mar_stat_b + phys_work_b + incomearealow, na.action = na.omit, data = exc97)

#summary(no97)

perc.diff <- (exp(coefficients(no97)[2:5])-1)*100

MD <- coef(no97)[2:5]

SE <- summary(no97)["se"]

SE <- as.data.frame(SE)

SE[2:5,]

marginoferror <- 1.96*SE[2:5,]

lwr <- (exp(MD - marginoferror)-1)*100

upr <- (exp(MD + marginoferror)-1)*100

No97 <- (round(cbind(perc.diff, lwr,upr), 1))


#6.	Include diet and leisure time physical activity (dropping 2012)
```

```
exc2012 <- filter(std4, study!=2012)

no2012 <-gam(log_crp ~ Q58 + s(age_b) + study + area+ edulev_b +smoking_b+  totalalc_week_cat_b_fin +
income_b +etsc_b_fin+ mar_stat_b + phys_work_b + incomearealow+  meat_b + fish_b + rawveg_b + fruit_b +
physact_w5a2_b, na.action = na.omit, data = exc2012)

#summary(no2012)

perc.diff <- (exp(coefficients(no2012)[2:5])-1)*100

MD <- coef(no2012)[2:5]

SE <- summary(no2012)["se"]

SE <- as.data.frame(SE)

SE[2:5,]

marginoferror <- 1.96*SE[2:5,]

lwr <- (exp(MD - marginoferror)-1)*100

upr <- (exp(MD + marginoferror)-1)*100

No2012 <- (round(cbind(perc.diff, lwr,upr), 1))


#7.	Include road-traffic NO2

NO2 <-gam(log_crp ~ Q58 + s(age_b) + study + area+ edulev_b +smoking_b+  totalalc_week_cat_b_fin +
income_b +etsc_b_fin+ mar_stat_b + phys_work_b + incomearealow+ NO2_road , na.action = na.omit, data =
std4)

#summary(NO2)

perc.diff <- (exp(coefficients(NO2)[2:5])-1)*100

MD <- coef(NO2)[2:5]

SE <- summary(NO2)["se"]
```

~~~
SE <- as.data.frame(SE)

SE[2:5,]

marginoferror <- 1.96*SE[2:5,]

lwr <- (exp(MD - marginoferror)-1)*100

upr <- (exp(MD + marginoferror)-1)*100

NO2 <- (round(cbind(perc.diff, lwr,upr), 1))



Table4 <- as.data.frame(cbind(Maininflu_aug, Med,Disease,BMI, Lowcrp,Season, No97,No2012, NO2))

Table4



```
~~~
